# Home-administered transcranial direct current stimulation is a feasible intervention for depression: an observational cohort study

**DOI:** 10.3389/fpsyt.2023.1199773

**Published:** 2023-08-22

**Authors:** Leigh Charvet, Allan George, Erik Charlson, Matthew Lustberg, Amy Vogel-Eyny, Tehila Eilam-Stock, Hyein Cho, Pamela Best, Luis Fernandez, Abhishek Datta, Marom Bikson, Kamran Nazim, Giuseppina Pilloni

**Affiliations:** ^1^Department of Neurology, New York University Grossman School of Medicine, New York, NY, United States; ^2^The Arthur S. Abramson Department of Rehabilitation Medicine, Albert Einstein College of Medicine, New York, NY, United States; ^3^Research and Development, Soterix Medical, Inc., Woodbridge Township, NJ, United States; ^4^Department of Biomedical Engineering, The City College of New York, New York, NY, United States

**Keywords:** depression, non-invasive brain stimulation, telehealth, home-based tDCS, digital health, transcranial direct current stimulation (tDCS), major depressive disorder

## Abstract

Transcranial direct current stimulation (tDCS) is an emerging treatment for major depression. We recruited participants with moderate-to-severe major depressive episodes for an observational clinical trial using Soterix Medical's tDCS telehealth platform as a standard of care. The acute intervention consisted of 28 sessions (5 sessions/week, 6 weeks) of the left anodal dorsolateral prefrontal cortex (DLPFC) tDCS (2.0 mA × 30 min) followed by a tapering phase of weekly sessions for 4 weeks (weeks 7–10). The *n* = 16 completing participants had a significant reduction in depressive symptoms by week 2 of treatment [Montgomery–Åsberg Depression Rating Scale (MADRS), *Baseline*: 28.00 ± 4.35 vs. *Week 2:* 17.12 ± 5.32, *p* < 0.001] with continual improvement across each biweekly timepoint. Acute intervention responder and remission rates were 75 and 63% and 88 and 81% following the taper period (week 10).

## 1. Introduction

Transcranial direct current stimulation (tDCS) is a safe and well-tolerated method of non-invasive brain stimulation. When delivered in repeated applications over time, tDCS targeting the left dorsolateral prefrontal cortex (DLPFC) is effective in the treatment of depression (1, 2). While the majority of tDCS depression trials have required clinic-based treatment, tDCS is feasible and reliable for at-home treatment (3) and for use as telehealth (4).

tDCS can be provided reliably at home by rigorously developed remotely supervised tDCS (RS-tDCS) technology and protocols (4) for patients with depression (5). Key technology innovations include correct self-application headgear optimized for DLPFC (6), tDCS limited-total-energy (7) (tDCS-LTE), pre-saturated single-use electrodes with snap connectors, and remote dose control and monitoring software that can further include digital therapies synced to tDCS administration (8).

Advantages of tDCS delivered to patients at home (4) include providing the number of sessions needed for optimal clinical efficacy and many other advantages of telehealth [e.g., continued treatment during the COVID-19 pandemic (9)]. New York University Langone Health (NYULH) extensively developed and validated a remotely supervised tDCS integration with telehealth treatments for home-based clinical trials (3). In response to patient demand, in 2019, NYULH designated a tDCS service program as innovative care, accessible to patients in any U.S. location by providing tDCS equipment and using NYU Video Visit through Epic for remote supervision.

Our objective was to evaluate the clinical feasibility of tDCS delivered as a virtual health intervention to facilitate planning for a pivotal regulatory trial. Here, we enrolled patients with a current episode of major depressive disorder (MDD) of at least moderate severity. Participants completed the at-home tDCS intervention as an observational study of standard of care.

## 2. Methods

This trial was sponsored by Soterix Medical (NCT04781127) and was initiated following an FDA request for home-based interventions in the context of the COVID-19 pandemic. NYULH was the enrolling site. The study was approved by the Western Institutional Review Board (WIRB) and NYULH IRB.

### 2.1. Participants

All participants were enrolled for treatment through the NYULH tDCS Program (10), and all procedures were completed through HIPAA-compliant video visits (Epic) and data collection platforms [Soterix Medical ElectraRx (11) and REDCap (12)].

Participants were recruited through referring clinicians and national advertisements. Eligible participants met the DSM-V-TR criteria for a current episode of unipolar MDD at least 4 weeks in duration with moderate severity [≥20 on the Montgomery–Åsberg Depression Rating Scale, MADRS (13)] and with any current medications stable for ≥30 days. Participants were excluded if they were at current suicide risk [Columbia Suicide Severity Rating Scale, CSSR-S (14)] or met the criteria for current or recent psychotic disorder, alcohol or substance use disorder in the past 3 months, and/or current use of benzodiazepines. The study excluded participants with depression who were judged to be due to a primary neurological disorder, currently or planning to become pregnant, or having any implanted device or metal above the neck or skin lesions that would interfere with tDCS.

### 2.2. Procedure

Potential participants were required to complete an initial prescreen for eligibility and were informed of the tDCS clinical program. The full treatment cost was $750; participants who completed all sessions and assessments were reimbursed $25 per week for the completion of measures (up to $250 total). Interested participants then consented and completed a psychiatric interview with a licensed psychiatrist (EC) for diagnosis confirmation and treatment history including the Mini International Neuropsychiatric Interview (MINI) (15). The MADRS was administered and served as the baseline assessment for those continuing with the intervention.

Participants were shipped the tDCS equipment kit that included the preprogrammed Soterix Medical 1X1 mini-CT device, unlocked for each session using a one-time code; left anodal DLPFC headset (6) (anode was placed over F3 and cathode over F4, according to 10–20 EEG system); and single-use SNAPpad sponge electrodes [see Pilloni et al. for illustration of procedures (3)]. Participants were also provided with audio-guided mindfulness meditation tracks at the start of each tDCS session customized for this trial to synergize with tDCS-LTE to address depression [“10 Min Mind” by Monique Rhodes (16)].

### 2.3. Intervention

At the initial treatment visit, participants were trained on the use of the equipment, completed a tDCS tolerability test, and were guided through their first treatment session. Participants completed a total of 32 DLPFC tDCS sessions (2.0 mA × 30 minutes, with 30-s ramp up/down of the electrical current at the beginning and end of each stimulation session) paired with 10 minutes of guided mindfulness meditation at each session start, followed by 20 minutes of relaxing music. Informed by prior tDCS depression trials (1, 5), we defined acute intervention to be 5 days/week, over ~6 weeks (weeks 1–6), and followed by 4 weeks of once-weekly taper sessions (weeks 7–10). Each session was completed as a video visit (NYU Video Visits) with a tDCS program clinician connecting to provide clearance and the activation code and to ensure compliance.

The ElectraRx (Soterix Medical) online platform was used for daily sessions to play the mindfulness audio tracks, to report adverse events (AEs), and complete daily self-report ratings. All patients were monitored at each daily session for a report of an increase in suicidality (C-SSRS) or other risk factors by the study psychiatrist (EC) and the treatment study team, with a plan for emergency action measures in place.

### 2.4. Outcomes and analyses

The primary outcome measure was the change in MADRS score from baseline to intervention end, administered at screening/baseline (EC) and approximately every 2 weeks via phone interview with AVE, HC, LC, and TES. Safety was monitored by the C-SSRS and tDCS AE queries. The responder rate was defined as a ≥50% improvement in the MADRS score (17) and remission by MADRS score ≤ 10 (17). Secondary outcomes included the Quick Inventory of Depressive Symptomatology Self-Report (18) (QIDS-SR) and the Quality of Life Enjoyment and Satisfaction Questionnaire Short Form (19) (Q-LES-Q-SF).

Statistical analysis was conducted using SPSS (IBM; Version 26). A repeated measure ANOVA (RM-ANOVA) was used to test the effect of the within-subject factor of TIME (2-week interval time points). A significant main effect was followed by *post-hoc t*-tests with Bonferroni correction for multiple comparisons to test the difference at each of the 2-week interval time point comparisons. The significance level was set at 0.05.

## 3. Results

A total of *n* = 24 participants consented, and *n* = 16 completed the intervention. Those who did not complete the study were because of failing to meet full eligibility criteria (*n* = 1), lost to follow-up post-screening visit (*n* = 1), and the discontinuation of treatment (*n* = 6) following one to nine tDCS sessions were due to: unrelated medical events (*n* = 1), treatment cost (*n* = 1), time (*n* = 1), and perceived lack of benefit (following completion of 4, 5, and 7 sessions, respectively).

### 3.1. Demographic and clinical features

Completed participants comprised 12 women and 4 men aged between 26 and 67 (mean 45 ± 13) years, identifying as White (*n* = 14), Asian (*n* = 1), and mixed race (*n* = 1). Participants had prior trials of antidepressant medications (*n* = 16), ketamine (*n* = 1), and electroconvulsive treatment (*n* = 1). All but one participant (*n* = 15) was on stable (>30 days) antidepressant medication at enrollment.

### 3.2. Study outcomes

There were no serious or treatment-limiting AEs caused by the tDCS intervention. No participant experienced an increase in depression or suicidality that warranted treatment discontinuation or additional intervention.

**Figure 1:**
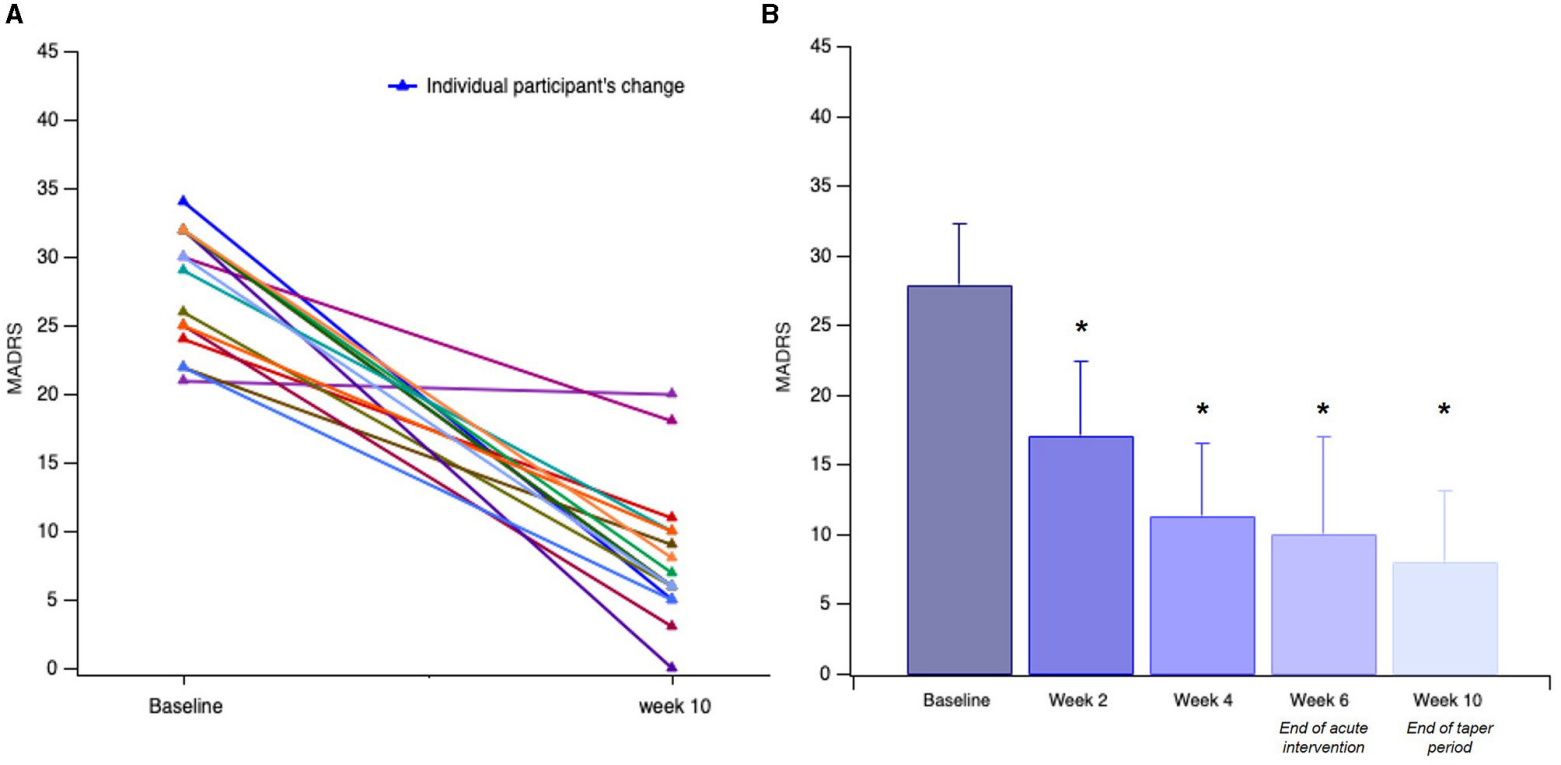
Participants' (*n* = 16) change in MADRS. **(A)** Individual change in MADRS from screen/baseline to end of taper intervention (week 10). **(B)** Linear change in mean scores for depression severity over time, assessed at 2-week intervals. Error bars indicate the standard deviation (SD) of the mean. Asterisks indicate significant improvement compared to the baseline (*p < 0.001).

We found a significant effect of the *TIME* [*F*_(1, 15)_ = 52.21, *p* < 0.001]. The participants had a mean treatment MADRS reduction of 18.81 ± 8.56 points (Figure 1A, *p* < 0.001). As shown in Figure 1B, the participants had a significant clinical response measured by the MADRS by Week 2 (*p* < 0.001), with continued improvement across the subsequent 2-week measures: *Baseline*: 28.00 ± 4.35, *Week 2:* 17.12 ± 5.32, *Week 4*: 11.38 ± 5.21, *Week 6 (end of acute intervention)*: 10.12 ± 6.97, and *Week 10 (end of taper sessions)*: 8.13 ± 5.07. Acute intervention (week 6) responder and remission rates were 75 and 63%, reaching 88 and 81% by the end of the taper sessions (week 10).

These improvements were mirrored by significant decreases in self-reported depressive symptoms [QIDS (18), 12.25 ± 4.99 vs. 7.50 ± 4.63, *p* < 0.001] and significant improvements in self-reported quality of life [Q-LES-Q-SF (19), 47.85 ± 11.00 vs. 63.87 ± 12.95, *p* < 0.001] at the end of taper period (week 10).

## 4. Discussion

We found the home-based tDCS depression intervention to be feasible and well-tolerated, resulting in a significant clinical benefit for individuals experiencing moderate and severe unipolar depression. Findings are overall consistent with the Level A evidence of tDCS as an effective treatment for depression (20) and supports the feasibility of at-home treatment with remote supervision (5). These findings will inform the design of the next step of home-based sham-controlled trials to further evaluate its effectiveness and inform dosing regiments.

Given the continual improvement across the 28-session acute intervention time points and further following the subsequent 4 weekly taper sessions, at-home delivery is particularly important to ensure that tDCS sessions are delivered at the necessary frequency and dose for optimal clinical effect. We experienced a relatively high rate of participant dropouts, attributed to a range of reasons, and most occurred before or early in the intervention period. Of note, the three participants who discontinued due to lack of perceived response completed ≤ 7 tDCS sessions, which is considered well below the threshold for expected improvement (2). While these participants may have been retained if there was the requirement to attend in-person vs. home-based visits, no reason for discontinuation was attributable to home-based treatment delivery. Instead, our observed attrition may be consistent with treatment trials of depression in general (21). Furthermore, we continue to find higher-than-expected retention rates across uses in tDCS clinical trials using home-based delivery (3).

All participants had tDCS accompanied by mindfulness meditation and music audio serving to produce a consistent “brain state” within and across the participants during the treatments. While the particular version of mindfulness that we used here (the “10 Min Mind”) has not been studied in previous trials, there is mixed evidence to date for the benefit of similar audio-guided mindfulness tracks alone (22). The separate or combined effect of mindfulness, as well as music, together with tDCS, cannot be determined from these findings. Importantly, a recent review found that tDCS can augment the benefits of mindfulness-based interventions, including reducing depression (23).

Our sample would meet the criteria for adjunctive treatment in the context of treatment-resistant depression (24), given the majority of participants (15 of 16) were on stable concurrent antidepressant medication for >6 weeks. We included patients stable on medication as this characterizes the majority of people living with depression and additionally avoids possible withdrawal symptoms in advance of trial participation (25). However, tDCS has previously been found effective as monotherapy and in milder depression as well (1, 2). As our tDCS service requires self-payment for treatment, our participants were not representative of the socioeconomic spectrum of people with depression.

These results strongly support the next step of larger multi-center home-based randomized controlled trial (RCT). Future trials will help determine the patients who are most likely to respond to tDCS, alone or in combination with other therapies.

## Data availability statement

The raw data supporting the conclusions of this article will be made available by the authors, without undue reservation.

## Ethics statement

The study involving humans was approved by Western Institutional Review Board (WIRB). The studies were conducted in accordance with the local legislation and institutional requirements. The participants provided their written informed consent to participate in this study. No potentially identifiable images or data are presented in this study.

## Author contributions

LC, AD, MB, KN, and GP designed the study and its intervention components. Participants were recruited by EC and LC, as well as through self-referrals to the clinical tDCS service. AG and ML completed prescreening and enrollment, while LC and EC completed the screening process and administration of the interview-based measures. LC, TE-S, AV-E, and HC completed the interview-based outcome measures. AG, ML, TE-S, PB, and LF administered the daily video visits for the intervention. The data were entered by AG and analyzed by LC and GP. LC, AD, MB, and GP interpreted the results. All authors critically revised the manuscript and approved the final version.

## Supplementary material

Supplementary Figure 1 Participants' (*n* = 16) change in MADRS. Individual and group change in MADRS over time, assessed at two-week intervals.
