# Supplementary figures and images for: Home-administered transcranial direct current stimulation is a feasible intervention for depression: an observational cohort study
Source: Front Psychiatry. 2023 Aug 22;14:1199773. doi: 10.3389/fpsyt.2023.1199773 (PMC10477781; doi:10.3389/fpsyt.2023.1199773)

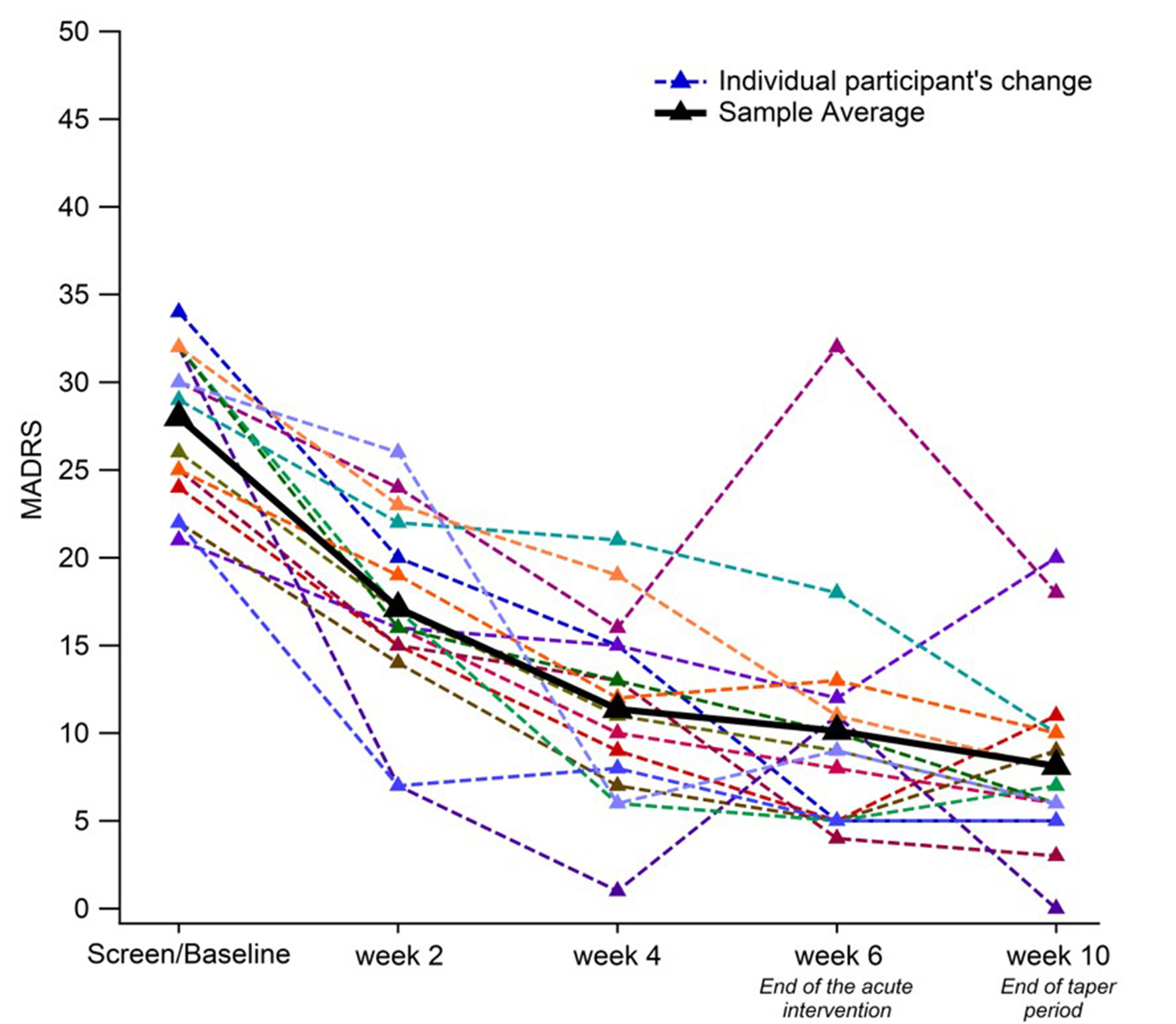

Supplement: Supplementary Figure 1 — Participants' (n = 16) change in MADRS. Individual and group change in MADRS over time, assessed at two-week intervals. [file Image_1.jpg]
